# Activation of B1 B cells by *F. tularensis* atypical LPS depends on classical complement and C3a

**DOI:** 10.1371/journal.ppat.1013799

**Published:** 2025-12-17

**Authors:** Guilherme Melo, Carlos Henrique D. Barbosa, Elena Magrini, Cecilia Garlanda, Fabio Re

**Affiliations:** 1 Rosalind Franklin University of Medicine and Science, North Chicago, Illinois, United States of America; 2 IRCCS Humanitas Research Hospital, Milan, Italy; Universidade de São Paulo: Universidade de Sao Paulo, BRAZIL

## Abstract

*Francisella tularensis* (*Ft*), a Gram-negative bacterium that causes tularemia, possesses a non-inflammatory atypical LPS (LPS_*Ft*_) that is highly immunogenic through unknown mechanism. We previously showed that immunization with LPS_*Ft*_, a type 2 T-independent (TI) antigen, elicits protective LPS_*Ft*_-specific IgM (IgM_*Ft*_) and IgG3_*Ft*_ by B1 cells in a mechanism dependent on the IL-5 produced by innate lymphoid cells type 2 (ILC2). Here, we examined the role of complement in the B1 cells’ response against LPS_*Ft*_. *C3*^-/-^, *C1q*^-/-^ and *C4*^-/-^ mice immunized with LPS_*Ft*_ failed to produce IgM_*Ft*_ and IgG3_*Ft*_. In contrast, the response of *Cfb*^-/-^ and *Mbl1/Mbl2*^-/-^ mice was comparable to that of WT mice. Thus, activation of the classical complement cascade, but not the alternative or the Mannose Binding Lectin pathway, is required for activation of B1 cells and production of LPS_*Ft*_-specific antibodies. Complement activation generates the C3d fragment, which opsonizes antigens for recognition by complement receptor-2 (CR2), and the C3a and C5a anaphylatoxins. Our results show that C3d opsonized LPS_*Ft*_ and that the response to immunization was dependent on CR2 expression by B1 cells. Importantly, the response to LPS_*Ft*_ immunization was also drastically decreased in *C3ar1*^-/-^, but not in *C5ar1*^-/-^ mice. C3a induced IL-5 in ILC2, which supported B1 cells activation. Decreased antibody production in *C3ar1*^-/-^ and *Cr2*^*-/-*^ mice correlated with increased susceptibility to tularemia. Together, these results demonstrate that the high immunogenicity of LPS_*Ft*_ depends on two effector mechanisms triggered by activation of the classical complement pathway: 1) tagging of LPS_*Ft*_ with C3d fragment, leading to its interaction with CR2 expressed by B1 cells; 2) production of the anaphylatoxin C3a that stimulated IL-5 secretion by ILC2. Our study increases our understanding of the B1 cells’ response to TI-2 antigens and identifies two complement effector mechanisms that can be harnessed for therapeutic interventions.

## Author summary

The lipopolysaccharide (LPS) of the bacterium *Francisella tularensis* strongly stimulates B cells for antibody production independently of T cell help through unknown mechanism. In the present study we examined the role of the complement cascade in this process. We found that production of antibodies against this LPS depends on activation of the classical complement pathway but not the MBL-dependent lectin or the alternative pathways. Following complement activation, LPS became tagged with the C3d complement fragment leading to its interaction with the complement receptor CR2 expressed by B cells. Complement activation also resulted in production of the anaphylatoxin C3a that was required for B cells activation, possibly through induction of IL-5 by innate lymphoid cells 2. Our study increases our understanding of the B cells’ response to T-independent antigens and identifies two complement effector mechanisms that can be harnessed for therapeutic interventions.

## Introduction

Antibodies produced by B cells are the most powerful effector mechanism of adaptive immunity. The B cell receptor (BCR) has the ability to directly recognize linear epitopes or conformational epitopes leading to B cells activation. Optimal activation of B cells and antibody production, particularly against protein antigens, often requires additional stimulation provided by T helper cells and other cell types in the form of cytokines and co-stimulatory molecules (reviewed in [1]). However, certain antigens can activate B cells in absence of T cell help. These T-independent (TI) antigens can be classified in two distinct groups (reviewed in [2,3]). TI-1 antigens, like the Gram-negative bacteria LPS, have the ability to simultaneously activate the BCR and innate immune receptors expressed by B cells, like the Toll-like Receptors (TLR), delivering two sufficiently strong signals for full activation of B cells and differentiation into plasma cells. These responses tend to be polyclonal and not antigen-specific. B cells activation by TI-2 antigens, in contrast, is believed to depend on the highly repetitive structure of these molecules that leads to clustering of numerous BCR in absence of innate immune stimulation. Whether other co-stimulatory signals are required for the response against TI-2 antigens is not clear. Evidence that the two signals model can apply to TI-2 antigens has been obtained for synthetic molecules *in vitro* [4] but not for microbial TI-2 antigen *in vivo* (reviewed in [5]). This is the issue the present study set out to determine.

B1 cells differ from the classical B2 cells for their developmental origin, are localized in the pleural and peritoneal cavity, have the ability to self-renew, and efficiently recognize and respond to TI antigens (reviewed in [2,6,7]). B1 cells are the primary source of natural antibodies, which tend to recognize TI antigens like capsular polysaccharides and self-antigens [8], but they also respond to pathogen-derived antigens, producing IgM and IgG3 isotypes that protect against various infections [9].

The complement system is a major effector mechanism of innate immunity and can be activated through the antibody-dependent classical pathway, the lectin pathway, or the alternative pathway [10]. Activation of the complement proteolytic cascade results in cleavage of the C3 component into the anaphylatoxin C3a and the C3b fragment that is covalently bound to pathogens or antigens, acting as an opsonin for improved phagocytosis. As the cascade continues, cleavage of the C5 component generates the C5a anaphylatoxin and triggers the assembly of the Membrane Attack Complex (MAC) on the pathogens surface leading to lysis.

Complement activation affects the immune response against both T-dependent and independent antigens at many different levels [11,12]. Antigens tagged with C3b, and the breakdown product C3d, interact with the CR2 complement receptor expressed by B cells drastically lowering the activation threshold [13,14]. C3d-modified antigens are also captured and retained by CR2-expressing Follicular Dendritic Cells (FDC) [15] and marginal zone macrophages [16] creating an antigen depot that allows prolonged and consistent B cells stimulation. One additional function of C3d-tagging is to signify the microbial origin of the antigen, a requirement that may be particularly important for TI-2 antigens due to the absence of DC-T helper cells involvement, which provides this information for T-dependent antigens. The C3a and C5a anaphylatoxins are strongly proinflammatory and, through activation of dendritic cells, macrophages, and T helper cells, are known to modulate cellular and humoral immunity at various levels [17–20]. Their involvement in the B cells response to TI antigens, however, is less well characterized.

*Francisella tularensis* (*Ft*) is a Gram-negative bacterium that causes tularemia [21]. *F. tularensis* live vaccine strain (LVS) is not pathogenic for healthy humans but causes a similar disease in mice and is widely used as an animal model for tularemia. Protection against tularemia depends on several effector mechanisms of innate and adaptive immunity, including antibodies [22–24].

The interaction of *Ft* with the complement system has been studied primarily *in vitro* by several groups (reviewed in [25]). It has been shown that *in vitro* both virulent Schu S4 strain and LVS activate the classical complement through natural antibody leading to opsonization by C3b [26–30]. Opsonization was required for efficient uptake and infection of myeloid cells. Complement receptors CR1, CR3, and CR4 mediated this uptake. Phagosome escape of opsonized bacteria was temporarily delayed but eventually this resulted in increased macrophage death [29]. Despite activation of the early steps of complement cascade, *Ft* evades complement-mediated lysis by binding factor H on its surface, thereby inhibiting assembly of the membrane attack complex [31]. Another mechanism through which *Ft* can exploit complement to its own advantage is by inhibiting TLR2 and inflammasome activation [32,33]. Taken together, these *in vitro* studies indicate that *Ft* has the ability to hijack the complement system for increased infectivity and to inhibit the TLR-mediated inflammatory response and the terminal complement-mediated lysis.

Whether these *in vitro* observations reflect what is happening in the infected host or have any relevance for the outcome of the infection and whether complement plays any protective role against *Ft* is not clear. In fact, the *in vivo* role of complement in tularemia has received little attention. Using a mouse model of tularemia, one study showed that C3-deficient mice had similar survival rate as WT mice after infection with LVS and that the prophylactic protection conferred by passive transfer of immune serum did not require complement [34]. This result is surprising and would suggest that potentially protective effector mechanisms of complement (anaphylatoxin-mediated inflammation and humoral immunity stimulation) may be counteracted by C3b opsonization of bacteria, which increases infectivity, and by the bacterium’s resistance to lysis. However, the present study shows that this is not the case and, in fact, complement activation is protective against *Ft*.

In contrast to the LPS of most Gram-negative bacteria, *Ft* LPS (LPS_*Ft*_) does not activate TLR or inflammasome and does not possess proinflammatory activities [35–37]. Accordingly, LPS_*Ft*_ should be classified as a TI-2 antigen. Antibodies against LPS_*Ft*_ protect from tularemia [24,34] and we and others have shown that they are produced by B1 cells [38–41]. We have also shown that activation of B1 cells by LPS_*Ft*_ depends on IL-5 produced by innate lymphoid cell type 2 (ILC2) that have been activated by IL-25, thus identifying an IL-25-ILC2-IL-5 axis critical for B1 cells response to LPS_*Ft*_ [42]. The ability of LPS_*Ft*_ to strongly activate B1 cells in absence of TLR stimulation is remarkable and suggests that it may activate other innate immune pathways delivering a second signal for B1 cells activation.

Here we show that classical complement activation plays a protective role in tularemia and it is critical for B1 cells activation by LPS_*Ft*_. We show that LPS_*Ft*_ becomes tagged with C3d complement fragment leading to its interaction with complement receptor CR2 on B1 cells reinforcing BCR signaling (signal 1). Moreover, the anaphylatoxin C3a, but not C5a, was also shown to be required for optimal response of B1 cells likely through the stimulation of IL-5 production by ILC2 (signal 2). These results significantly advance our understanding of the role of complement in tularemia and of the mechanism responsible for B1 cells activation by TI-2 antigens, pointing to potential approaches to improve vaccination with this type of antigens.

## Results

### B1 cells’ response to immunization with LPS_*Ft*_ depends on complement

**Fig 1:**
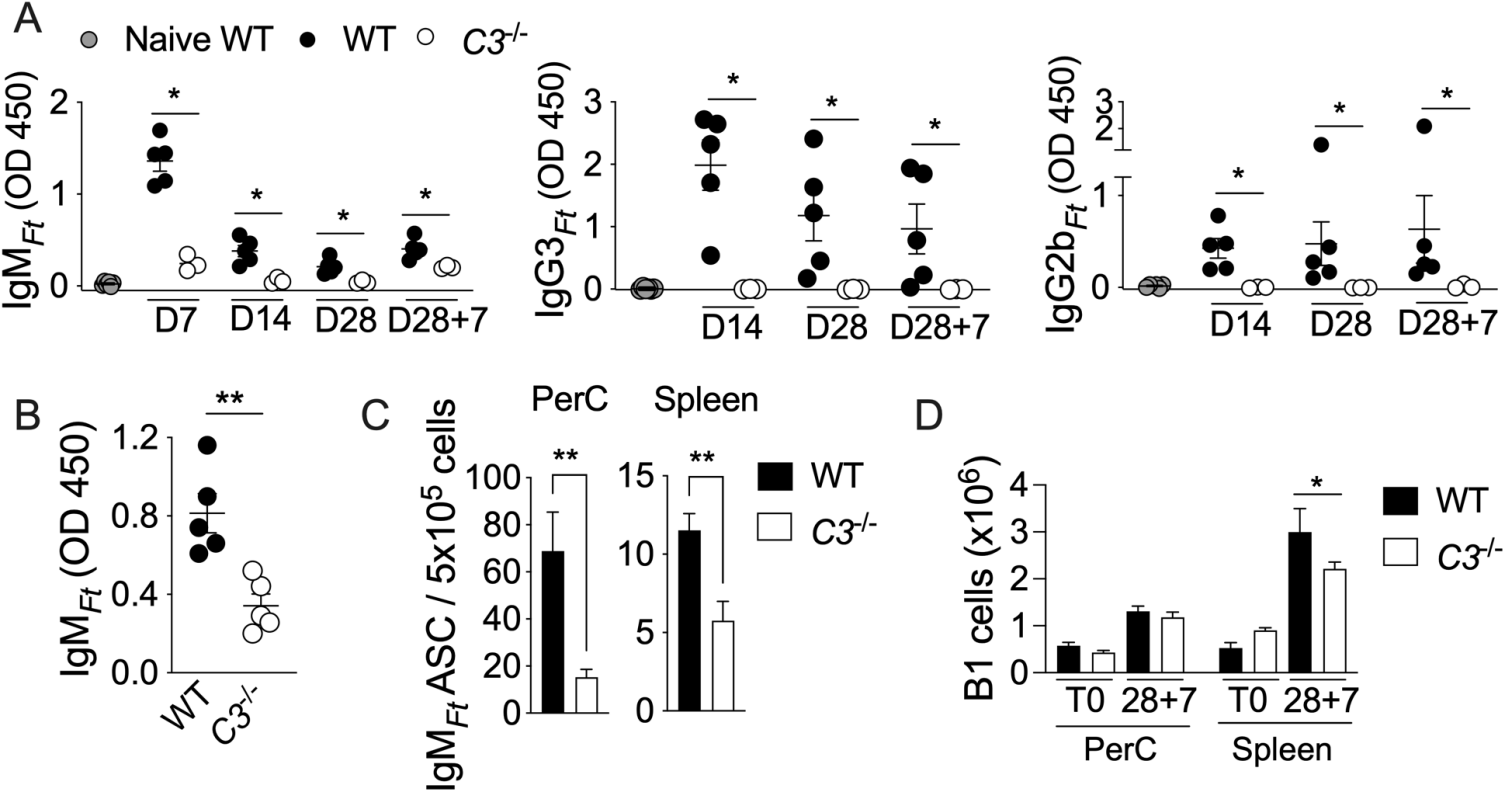
B1 cells’ response to immunization with LPS_*Ft*_ depends on complement. Wild type or *C3*^*-/-*^ mice were immunized with LPS_*Ft*_ and received a booster shot on day 28 (D28). LPS_*Ft*_-specific antibody levels were measured in serum at indicated time points **(A)** or in the peritoneal cavity wash at D28 + 7 **(B)**. **(C)** IgM_*Ft*_ ASC numbers were measured by ELISPOT in peritoneal cavity or spleen at D28 + 7. **(D)** Total number of B1 cells were analyzed by flow cytometry in peritoneal cavity or spleen at indicated times. One representative experiment of 3. Data are expressed as mean ± SEM. Mann-Whitney U test **(A–C)**; Two-way ANOVA with Tukey’s multiple comparison test **(D)**. *p* < 0.05*, *p* < 0.01**.

In order to determine the mechanism by which LPS_*Ft*_ activates B1 cells for production of antigen-specific antibodies, mice deficient in the C3 complement component were immunized with LPS_*Ft*_. Sera were obtained at different time points post-immunization (p.i.) and seven days after the booster shot (D28 + 7) to measure antibodies production. As shown in Fig 1A, the level of LPS_*Ft*_-specific IgM (IgM_*Ft*_) in WT mice peaked at day 7 and taper off thereafter. IgG3_*Ft*_ and IgG2b_*Ft*_ production was slower, peaking on day 14 and remaining elevated for a few weeks. In contrast, production of all three isotypes was significantly decreased or absent in *C3*^*-/-*^ mice. As expected for a TI antigen [43], the secondary immunization did not elicit a booster effect for all isotypes in both mouse strains. The level of IgM_*Ft*_ in peritoneal wash after booster shot was also lower in *C3*^*-/-*^ mice (Fig 1B). LPS_*Ft*_-specific IgG1, IgA, or IgE were not detectable at any time point (S1 Fig). Upon recognition of their cognate antigen, B1 cells migrate to the spleen where they differentiate into antibody secreting cells (ASC) [44]. The number of IgM_*Ft*_-producing ASC was drastically decreased in spleen and peritoneal cavity of *C3*^*-/-*^ mice compared to WT mice (Fig 1C). The total number of peritoneal B1 cells at steady state was comparable in both strains. However, upon immunization, *C3*^*-/-*^ mice showed significantly reduced total number of B1 cells in spleen (Fig 1D). These results show that B1 cells activation, migration to spleen, and differentiation into ASC in response to LPS_*Ft*_ immunization depends on complement activation, a result that agrees with the known effect of complement on B cells activation [12]. Interestingly, the response to vaccination with NP-ficoll, a synthetic TI-2 antigen, was not affected by C3 absence (S2 Fig), as previously shown [45].

### The classical complement pathway is required for B1 cells activation

**Fig 2:**
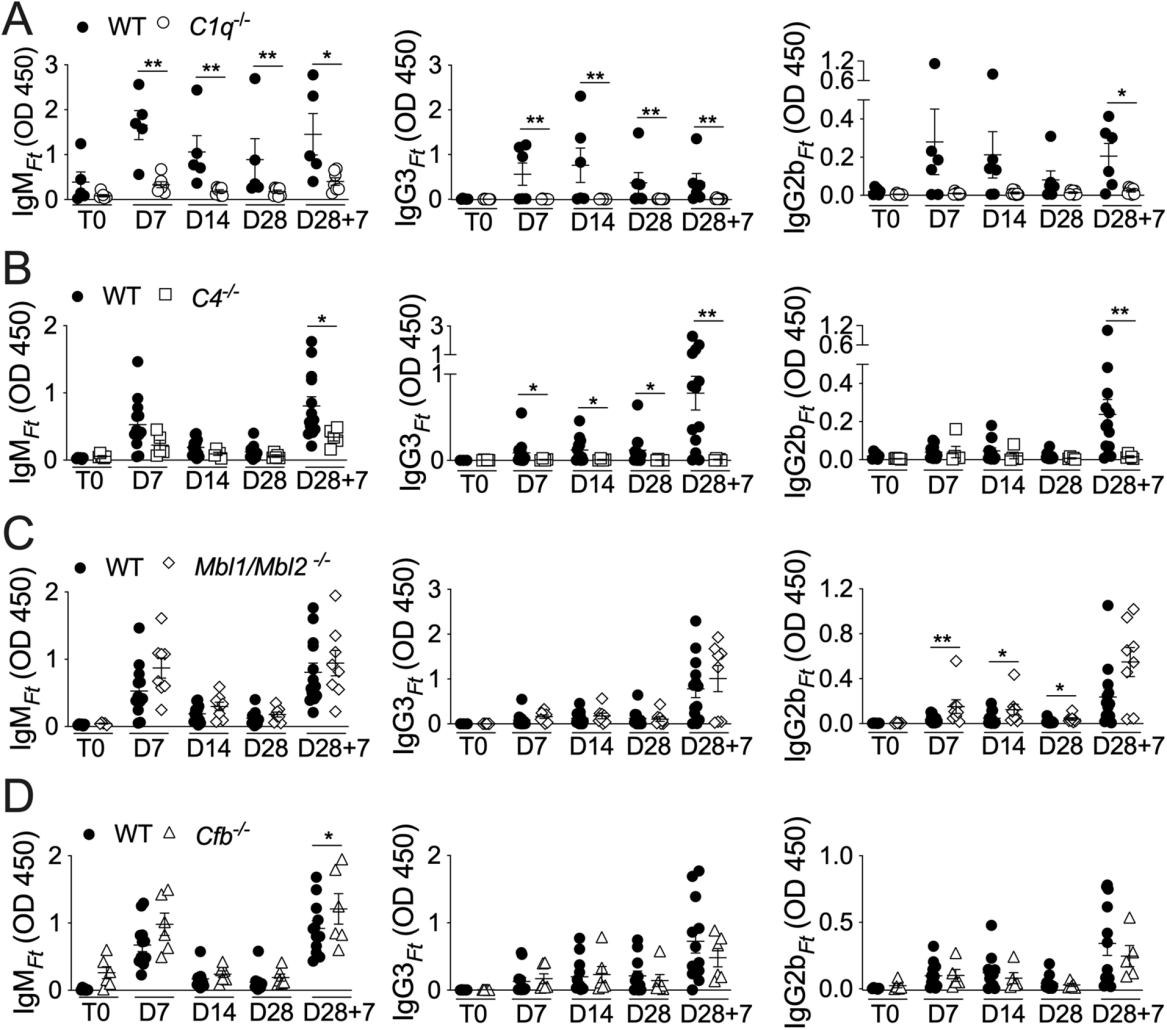
The complement classical pathway is required for B1 cells activation by LPS_*Ft*_. Wild type or *C1q*^*-/-*^, *C4*^*-/-*^, *Mbl1/Mbl2*^*-/-*^, or *Cfb*^*-/-*^ mice were immunized with LPS_*Ft*_ and IgM_*Ft*_, IgG2b_*Ft*_, IgG3_*Ft*_ levels were measured in serum at indicated time points. Data are expressed as mean ± SEM. Mann-Whitney U test. *p* < 0.05*, *p* < 0.01**.

To determine which complement pathway is required for B1 cells activation, mice deficient in each of the three complement pathways were immunized and antibody levels were measured in sera at different time points. As shown in Fig 2A, 2B, production of LPS_*Ft*_-specific IgM, IgG3, and IgG2b was significantly inhibited in mice lacking the C4 or C1q classical complement pathway molecules. In contrast, the antibody production was not affected in mice deficient in the MBL-dependent lectin pathway (*Mbl1*^*-/-*^*/Mbl2*^*-/-*^) (Fig 2C) or the alternative pathway (*Cfb*^*-/-*^) (Fig 2D). These results show that production of LPS_*Ft*_-specific antibodies by B1 cells is dependent on activation of the classical complement pathway.

### IgM and IgG3 participate in the classical complement activation by LPS_*Ft*_

**Fig 3:**
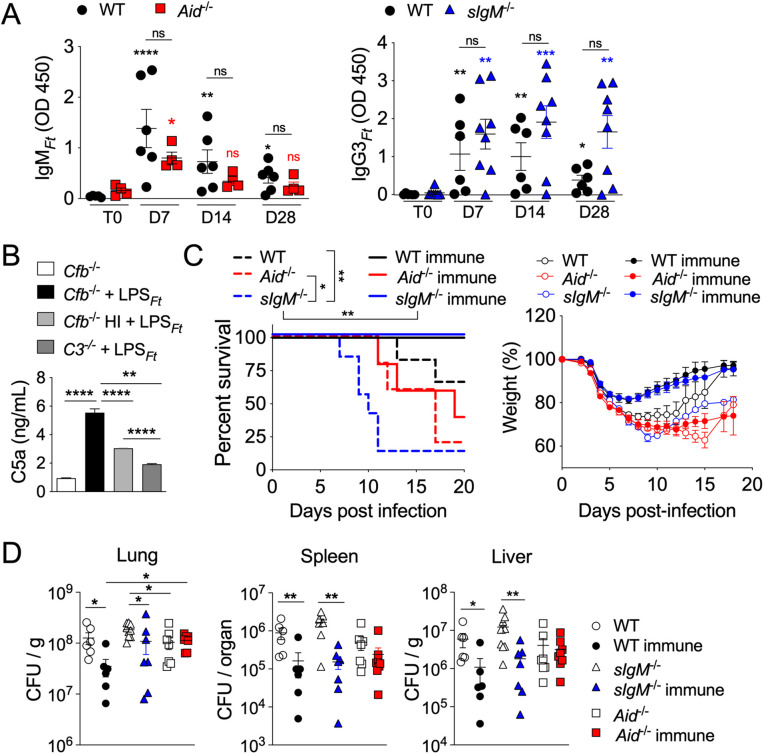
IgM and IgG3 participate to the classical complement activation by LPS_*Ft*_. **(A)** Wild type, *Aid*^*-/-*^, or *sIgM*^*-/-*^ mice were immunized with LPS_*Ft*_ and antibody levels were measured in serum at indicated time points. The asterisks (red or blue) denote, for each genotype, the comparison of the immunized vs the T0 naïve mice. **(B)** LPS_*Ft*_ was incubated with serum from *Cfb*^*-/-*^ or *C3*^*-/-*^ mice and generation of C5a was measured by ELISA. HI, heat inactivation. **(C)** Naïve or immunized WT, *Aid*^*-/-*^, and *sIgM*^*-/-*^ mice were infected with *Ft* LVS (4x10^3^ cfu) and survival and body weight were measured at indicated time points. **(D)** Bacteria burden in organs was measured in naïve or immunized mice 7 days after infection. One representative experiment of 5 (A) or 2 (D). Data are expressed as mean ± SEM. (A, D) Kruskal-Wallis test with Dunn’s multiple comparison test. (B) One-way ANOVA with Tukey’s multiple comparison test. (C) Gehan-Breslow-Wilcoxon test. *p* < 0.05*, *p* < 0.01**, *p* < 0.001*** (A).

IgM and IgG3 are potent activators of the classical complement pathway [10]. Natural IgM are present in naïve mice, tend to recognize capsular polysaccharides, and are made by B1 cells [8] suggesting this isotype may be responsible for complement activation during immunization with LPS_*Ft*_. To test the role of natural IgM in B1 cells’ primary response to LPS_*Ft*_ we immunized mice unable to secrete IgM (*sIgM*^*-/-*^) or *Aid*^*-/-*^ mice that cannot undergo class switch recombination. As shown in Fig 3A, the response of *Aid*^*-/-*^ mice, which can only secrete IgM isotype, was comparable to WT mice’s, supporting natural IgM’s role in complement activation by LPS_*Ft*_. Interestingly, the response of *sIgM*^*-/-*^ mice was also comparable (if not superior) to WT mice indicating that both natural IgM and IgG3 have the potential to contribute to the classical complement activation during the primary response to immunization. However, it should be noted that *sIgM*^*-/-*^ mice are known to have higher levels of IgG3 than WT mice [46]. Therefore, it is likely that in WT mice natural IgM are the main contributors to complement activation, as others have shown [30]. The superior ability of natural IgM to activate complement was shown by incubating LPS_*Ft*_ with serum obtained from naïve *Cfb*^*-/-*^ mice and measuring potent C5a generation by ELISA (Fig 3B). LPS from *F. novicida* was also able to activate complement while NP-ficoll did not (S3 Fig). Heat inactivated *Cfb*^*-/-*^ serum or *C3*^*-/-*^serum generated significantly reduced C5a amount. For this assay we used *Cfb*^*-/-*^ mice serum in order to avoid the high background due to spontaneous activation of the alternative pathway. An additional advantage was that naïve *Cfb*^*-/-*^ mice serum contained higher level of IgM_*Ft*_ than WT naïve serum but little IgG3_*Ft*_ (see Fig 2D). We next infected intranasally naïve or immunized WT, *sIgM*^*-/-*^, or *Aid*^*-/-*^ mice with *Ft* LVS and measured their survival and weight loss (Fig 3C). Naïve *sIgM*^*-/-*^ mice were more susceptible than naïve WT or *Aid*^*-/-*^ mice suggesting that IgM_*Ft*_ is an effective protective mechanism, as we previously showed [38]. However, the susceptibility of the immunized mice showed quite a different pattern: immunization completely protected WT and *sIgM*^*-/-*^ mice but had no effect on the survival of *Aid*^*-/-*^ mice. Body weight loss in the different mouse strains followed a similar pattern and correlated with their survival. Immunization significantly decreased the bacteria burden at day 7 in organs of WT and *sIgM*^*-/-*^ mice but not of *Aid*^*-/-*^ mice (Fig 3D). These results indicate that immunization with LPS_*Ft*_ protects from tularemia through IgG3_*Ft*_ while IgM_*Ft*_ play a more important role during the primary response to infection of naïve mice.

### The anaphylatoxin C3a, but not C5a, is required for B1 cells activation

**Fig 4:**
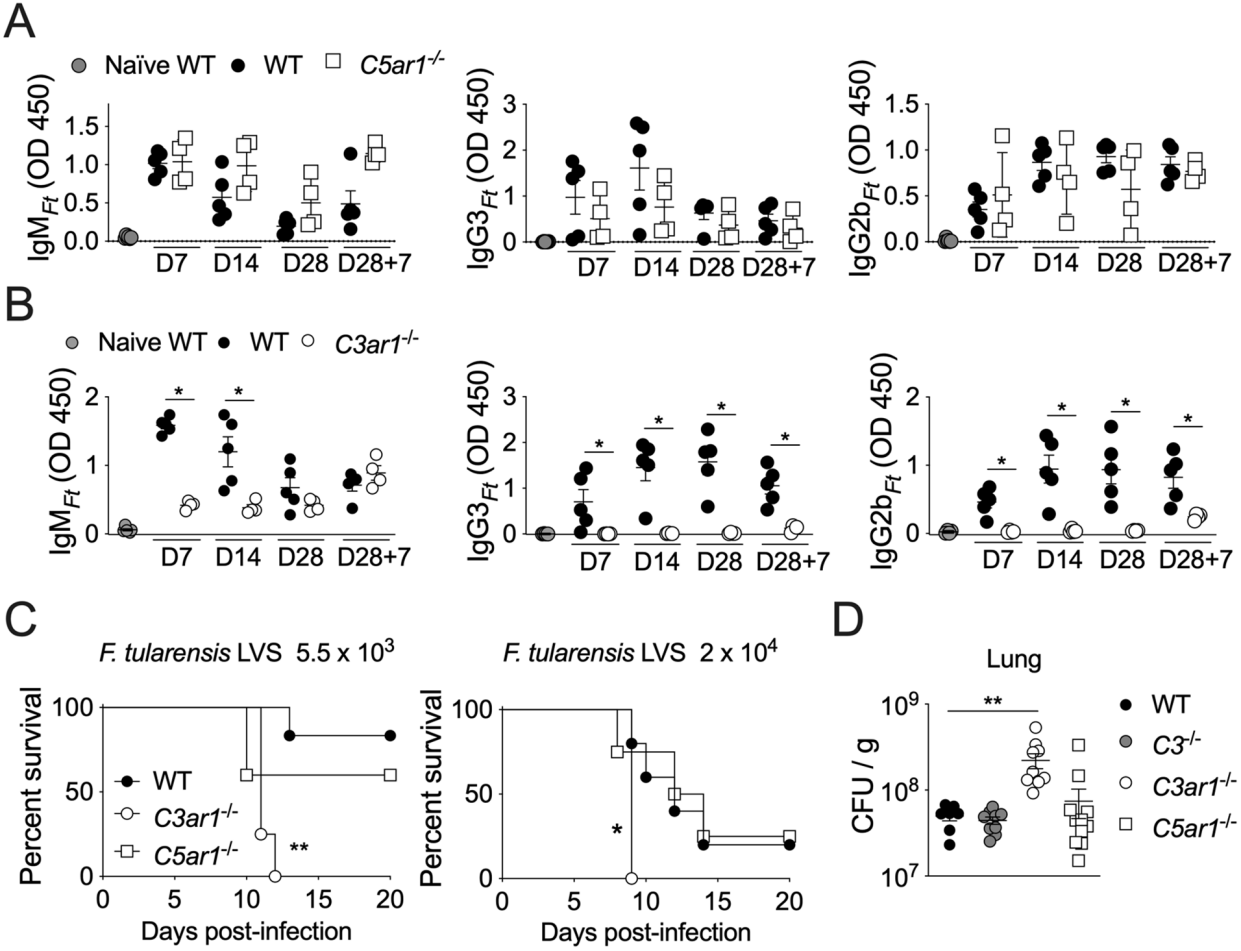
The anaphylatoxin C3a, but not C5a, is required for B1 cells activation. Wild type or *C5ar1*^*-/-*^ **(A)** or *C3ar1*^*-/-*^ **(B)** mice were immunized with LPS_*Ft*_ and antibody levels were measured in serum at indicated time points. Wild type or *C3*^*-/-*^*, C5ar1*^*-/-*^, or *C3ar1*^*-/-*^ mice were infected with *Ft* LVS (5.5x10^3^ cfu or 2x10^4^ cfu) and **(C)** survival was measured at indicated time points or **(D)** lung bacteria burden was measured 7 days after infection. One representative experiment of 3 (A, B) or 2 (D). Data are expressed as mean ± SEM. (A, B) Mann-Whitney U test. **(C)** Gehan-Breslow-Wilcoxon test. **(D)** Kruskal-Wallis test with Dunn’s multiple comparison test. *p* < 0.05*, *p* < 0.01**.

We next investigated whether the anaphylatoxins C3a and C5a played any role in the response of B1 cells to LPS_*Ft*_ immunization. Their involvement in the humoral response has been investigated mostly for T-dependent antigen while their contribution to TI response is still unclear [17–19,47]. As shown in Fig 4A, production of LPS_*Ft*_-specific antibodies was not affected by absence of C5aR1. In contrast, the response of *C3ar1*^*-/-*^ mice to LPS_*Ft*_ immunization was significantly inhibited (Fig 4B). The number of peritoneal B1 cells at steady state was comparable among the three strains but significantly lower number of B1 cells migrated to the spleen after immunization of *C3ar1*^*-/-*^ mice. (S4 Fig).

The role of anaphylatoxins or complement in tularemia is not known. To clarify this issue, we intranasally infected WT, *C3*^*-/-*^, *C3ar1*^*-/-*^, or *C5ar1*^*-/-*^ mice with *Ft* LVS and measured their susceptibility. *C3ar1*^*-/-*^ mice showed significantly reduced survival compared to WT or *C5ar1*^*-/-*^ mice (Fig 4C) and had higher lung bacteria burden (Fig 4D). *C3ar1*^*-/-*^ mice also showed increased lung inflammation with high cytokine levels and myeloid cells infiltration (S5 Fig) likely due to the higher bacteria burdens. These results indicate that *C3ar1*^*-/-*^ mice are more susceptible to *Ft,* likely due to defective production of IgM_*Ft*_, although other mechanisms may also be involved.

### Activation of B1 cells depends on the interaction of C3d-tagged LPS_*Ft*_ with CR2

**Fig 5:**
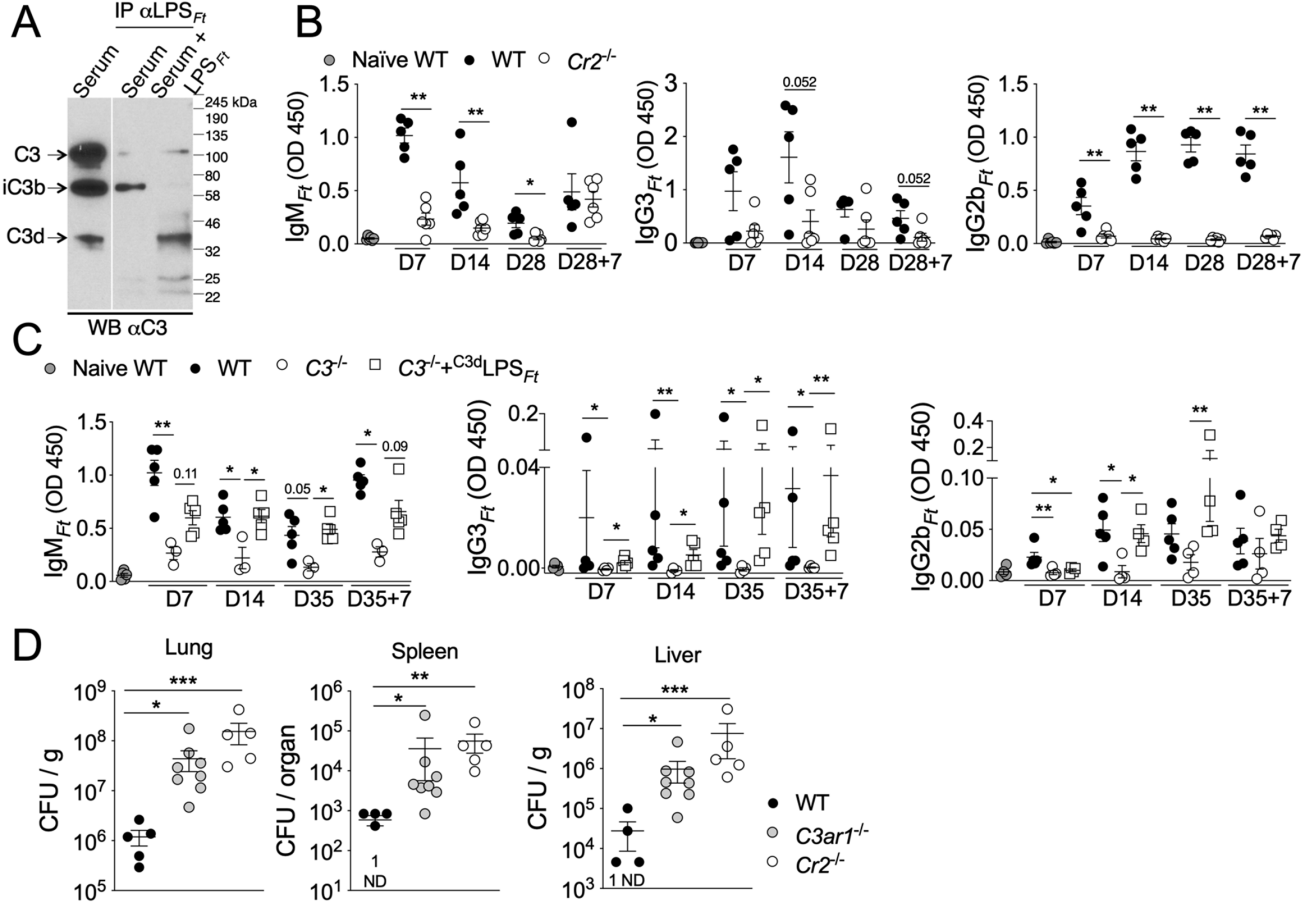
Activation of B1 cells depends on the interaction of C3d-tagged LPS_*Ft*_ with CR2. **(A)** LPS_*Ft*_ was incubated with fresh serum obtained from LPS_*Ft*_-immunized WT mice and immunoprecipitated with a mAb anti-LPS_*Ft*_ to demonstrate tagging with C3d complement fragment. **(B)** Wild type or *Cr2*^*-/-*^ mice were immunized with LPS_*Ft*_ and antibody levels were measured in serum at indicated time points. **(C)** Wild type or *C3*^*-/-*^ mice were immunized with LPS_*Ft*_ or with LPS_*Ft*_ tagged with C3d (^C3d^LPS_*Ft*_) and antibody levels were measured in serum at indicated time points. **(D)** LPS_*Ft*_-immunized WT, *C3ar1*^*-/-*^, or *Cr2*^*-/-*^ mice were infected with *Ft* LVS (4x10^3^ cfu) and bacteria burden in organs was measured 7 days later. One representative experiment of 2. Data are expressed as mean ± SEM. **(B)** Mann-Whitney U test. **(C, D)** Kruskal-Wallis test with Dunn’s multiple comparison test. *p* < 0.05*, *p* < 0.01**, *p* < 0.001***.

One main mechanism through which complement activation is known to enhance the humoral immune response is by tagging antigen with the C3d complement fragment [14,48]. Antigens tagged with C3d stimulate B cells through the CR2 complement receptor, drastically lowering the threshold of activation [49]. As shown in Fig 5A, LPS_*Ft*_ incubated *in vitro* with fresh serum obtained from LPS_*Ft*_-immunized WT mice became tagged with C3d suggesting that interaction with CR2 may be critically involved in LPS_*Ft*_ immunogenicity. This hypothesis was proved correct as CR2-deficient mice were significantly impaired in their response to immunization with LPS_*Ft*_ (Fig 5B). Importantly, and supporting the critical role of C3d-tagging, LPS_*Ft*_ tagged *in vitro* (as in Fig 5A) rescued the response to immunization of *C3*^*-/-*^ mice (Fig 5C). Confirming CR2 role in the response to LPS_*Ft*_ and the protective role of LPS_*Ft*_-specific antibodies, LPS_*Ft*_-immunized *Cr2*^*-/-*^ (and *C3ar1*^*-/-*^ mice) were more susceptible to intranasal infection with *Ft* LVS compared to immunized WT mice, showing significantly higher bacteria burdens in organs (Fig 5D). Interestingly, on the day of infection (D35), the most prominent LPS_*Ft*_-specific isotype was IgG3 (S6 Fig), again suggesting (as in Fig 3) that immunization protects primarily by inducing IgG3-switched B1 memory cells.

### CR2 expression on B1 cells is required for response to LPS_*Ft*_ immunization

**Fig 6:**
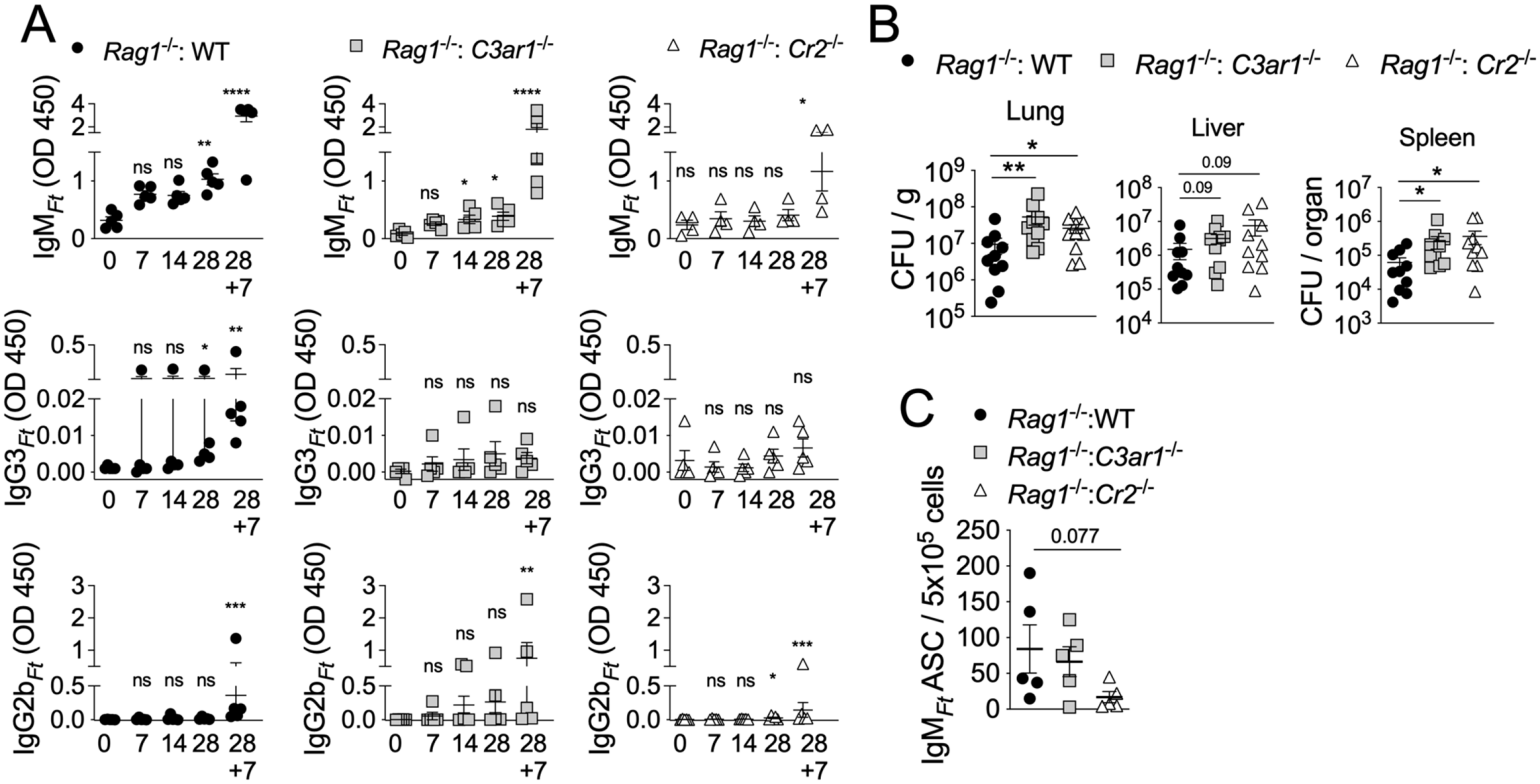
CR2 expression on B1 cells is required for response to LPS_Ft_ immunization. **(A)** *Rag1*^*-/-*^ mice reconstituted with WT, *C3ar1*^*-/-*^, or *Cr2*^*-/-*^ peritoneal B1 cells were immunized with LPS_*Ft*_ and antibody levels were measured in serum at indicated time points. The asterisks denote, for each genotype, the comparison of the immunized vs the T0 naïve mice. Immunized mice (D28 + 7) were infected with *Ft* LVS (4x10^3^ cfu) and bacteria burden in organs **(B)** or ASC in spleen **(C)** were measured 7 days later. One representative experiment of 2. Data are expressed as mean ± SEM. Kruskal-Wallis test with Dunn’s multiple comparison test. *p* < 0.05*, *p* < 0.01**, *p* < 0.001***, *p* < 0.0001****.

To determine whether B cells-intrinsic expression of CR2 or C3aR1 was important for the response to LPS_*Ft*_, peritoneal B1 cells were purified from WT, *C3ar1*^*-/-*^, or *Cr2*^*-/-*^ mice and adoptively transferred into *Rag1*^*-/-*^ mice. Four weeks post-transfer, the B1 cells repopulated the peritoneum and spleen (S7 Fig) and mice were immunized with LPS_*Ft*_ to measure antibody levels and ASC. As shown in Fig 6A, mice reconstituted with WT or C3aR1-deficient B1 cells were able to respond to immunization and produced IgM_*Ft*_. In contrast, mice reconstituted with CR2-deficient B1 cells were significantly impaired in their ability to produce IgM_*Ft*_ (which was induced only after the booster shot, D28 + 7). Interestingly, production of IgG3_*Ft*_ or IgG2b_*Ft*_ was severely affected in all adoptively transferred mice suggesting that bystander T helper cells or FDC functions, which are absent in *Rag1*^*-/-*^ mice, may be required for class switch recombination in B1 cells. The B1 cells-reconstituted mice were then infected (on D28 + 7) with *Ft* LVS and bacteria burden in organs (Fig 6B) and number of ASC (Fig 6C) were measured 7 days later. Consistent with the notion that immunization protects primarily through induction of IgG3_*Ft*_, mice reconstituted with CR2- or C3aR1-deficient B1 cells were more susceptible than mice reconstituted with WT B1 cells (which did produce IgG3_*Ft*_ after booster shot). Thus, CR2 expression on B1 cells is required for their primary response and IgM_*Ft*_ production while absence of C3aR1 expression on B1 cells had minimal effect on IgM_*Ft*_ production, suggesting that other cell types expressing C3aR1 may contribute to B1 cells’ activation by LPS_*Ft*_.

### C3a induces IL-5 expression by ILC2

**Fig 7:**
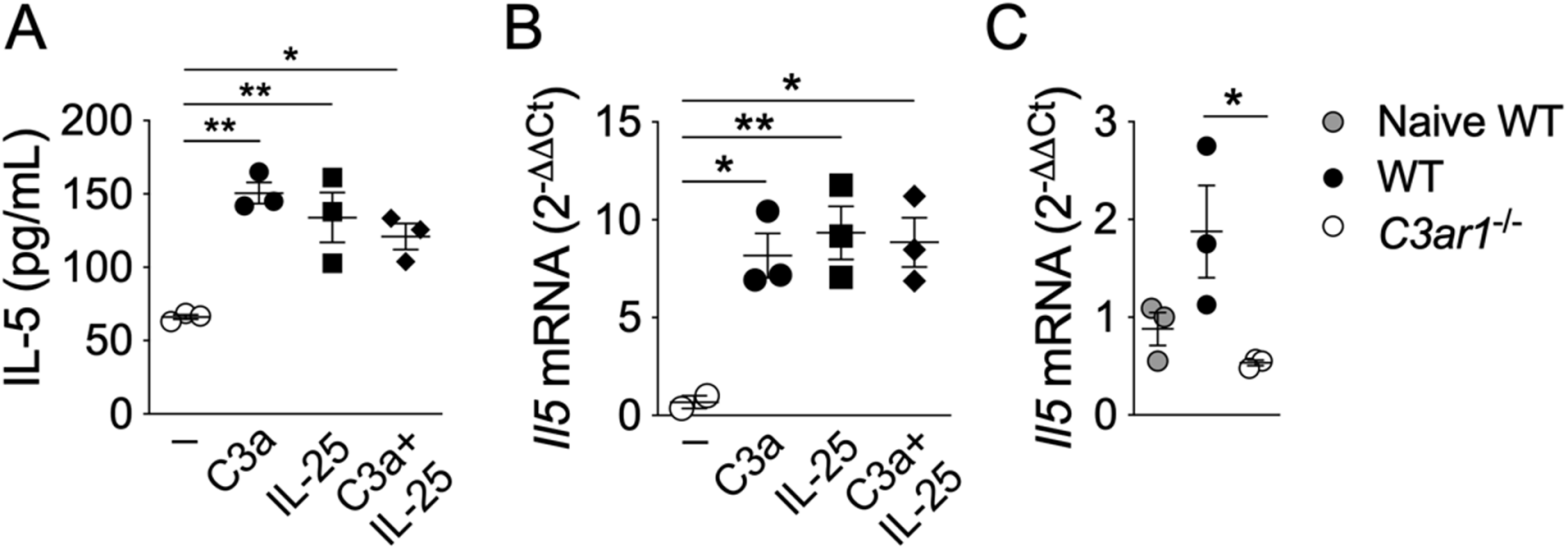
C3a induces IL-5 expression by ILC2. **(A, B)** ILC2 were purified from lung of WT mice and stimulated for 36 hours *in vitro* with C3a receptor agonist (2 μg/mL), IL-25 (100 ng/mL), or both. IL-5 was measured in conditioned supernatants by ELISA and *Il5* mRNA by RT-PCR. **(C)** Wild type or *C3ar1*^*-/-*^ mice were immunized intranasally with LPS_*Ft*_ and *Il5* mRNA was measured by RT-PCR in lung 18 hours post-immunization. Data are expressed as mean ± SEM. One-way ANOVA with Tukey’s multiple comparison test. *p* < 0.05*, *p* < 0.01**.

We previously demonstrated that activation of B1 cells during immunization with LPS_*Ft*_ depends on IL-5 produced by ILC2 [42]. Interestingly, C3a has been shown to stimulate cytokine production by ILC2 [50] suggesting that C3a may promote B1 cells activation through induction of IL-5 release by ILC2. To test this hypothesis ILC2 were purified from lung of WT mice and stimulated *in vitro* with C3a in presence or absence of IL-25, a known activator of ILC2. As shown in Fig 7A, 7B, IL-5 protein and mRNA were significantly induced by C3a even in absence of IL-25. Supporting the hypothesis that a role of C3a in the response to LPS_*Ft*_ is to induce IL-5 expression by ILC2 we observed (Fig 7C) that immunization with LPS_*Ft*_ induced IL-5 mRNA transcripts in lung of WT (as previously shown [42]) but not *C3ar1*^*-/-*^ mice. It is possible that C3a stimulates IL-5 production also in other cell types. Both C3a and C5a have been shown to regulate B cells survival through induction of the activating factor BAFF in neutrophils [47]. However, neutrophils depletion did not affect B1 cells response to LPS_*Ft*_ immunization (S8 Fig). Taken together these results indicate that the effect of C3a on B1 cells activation may be mediated through stimulation of IL-5 secretion by ILC2.

## Discussion

The innate immune response provides a plethora of signals that activate and inform adaptive immunity [51]. In the context of an infection, microbial products recognized by PRR generate an abundance of these signals in the form of cytokines, cell surface co-stimulatory molecules, and other inflammatory mediators. However, during vaccination with purified antigens these signals can be generated only if the antigen has the intrinsic ability to stimulate innate immunity. Otherwise, adjuvants must be included in the vaccine formulation.

Several TI-2 synthetic or microbial antigens have the ability to activate complement [5,10,52,53], though this requirement for B1 cell activation has not been extensively examined. Although LPS_*Ft*_ does not activate TLR or inflammasome and it is not proinflammatory [35–37], it is strongly immunogenic. Our results show that its ability to stimulate B1 cells’ antibody production is dependent on activation of the classical complement pathway (Figs 1, 2). As a result, LPS_*Ft*_ becomes tagged with C3d fragment (Fig 5) leading to its interaction with CR2 expressed on B1 cells (Fig 6) potentiating BCR signaling (signal 1). Concomitantly, C3a generation delivers a second signal for B1 cells activation (Fig 4) that is likely mediated by stimulation of IL-5 production by ILC2 (Fig 7). This complement-dependent activation of B1 cells triggers production of IgM_*Ft*_ and IgG3_*Ft*_ that protect from tularemia (Fig 3).

Several groups have examined the interaction of *Ft* with complement *in vitro* and revealed how the bacterium is able to evade MAC-mediated lysis and exploit opsonization to increase infectivity (reviewed in [25]). The majority of these studies were performed *in vitro* and would support the conclusion that complement activation may not be a protective response in tularemia. However, the role of complement during infection of the mammalian host has not been thoroughly examined *in vivo* and it is still unclear whether complement effector mechanisms, like anaphylatoxin-mediated inflammation and humoral immunity stimulation, could play a protective role.

Our study is the most thorough examination to date of the complement’s role in tularemia using an animal model. We showed for the first time that mice deficient in C3aR1 and CR2, but not C3 or C5aR1, were more susceptible to tularemia (Figs 4, 5). We also revealed that B1 cells’ activation by LPS_*Ft*_ and production of protective LPS_*Ft*_-specific antibodies depended on activation of the classical complement pathway (Fig 2). These results indicate that the impaired antibody production in complement deficient mice is likely responsible for their increased susceptibility, though other mechanisms may also be involved. Future studies will explore this possibility.

The observation, by us and others [34], that WT and *C3*^*-/-*^ mice have similar susceptibility to tularemia while *C3ar1*^*-/-*^ mice are clearly less resistant (Figs 4, 5) is puzzling. One possible explanation is that complement activation may simultaneously trigger protective responses, like C3d-opsonization of antigen and activation of ILC2 by C3a, but also deleterious ones, like increased infectivity of C3b-opsonized bacteria [27–29,31].

While we and others have shown that anti-LPS_*Ft*_ IgM produced by B1 cells are protective [38,39], it has also been shown that *Btk*^*-/-*^ mice, which lack B1a cells and IgM/IgG3, are more resistant to *Ft* infection [54] suggesting that B1a cells can have deleterious effect in tularemia. This phenotype was ascribed to B1a-released IL-10 that inhibited IL-12 and IFNγ production by macrophages and NK/NKT cells, respectively. We believe this result likely underscores the importance of the production of IL-12 and IFNγ and of a potent inflammatory response in the protection from tularemia. *Ft* has developed various strategies to counteract these responses (LPS structure modification, CR3-mediated inhibition of TLR stimulation). However, it is likely that, in absence of the anti-inflammatory function of B1a-derived IL-10, the regained proinflammatory cytokines production and effector functions of macrophage and NK/NKT cells provide a sufficiently strong protective advantage in *Btk*^*-/-*^ mice, even in absence of B1-derived protective antibodies. It should be mentioned that BTK deficiency does not affect conventional B2 cells to the same extent as B1a cells [55], allowing some residual T-dependent production of antibodies, other than IgM and IgG3, that can provide protection. An additional mechanism that can explain this surprising result is that absence of IgM/IgG3 in *Btk*^*-/-*^ mice would prevent classical complement activation and C3b opsonization of *Ft*, a step necessary for CR-3-mediated uptake and optimal infection of myeloid cells.

LPS_*Ft*_ has been shown to activate the classical pathway through natural IgM [30,56]. Interestingly, these antibodies were not able to bind directly the bacteria. Our results indicate that both natural IgM and IgG3 have the potential to support classical complement activation during the primary immunization with LPS_*Ft*_ (Fig 3). Although IgG3 is known to have the highest affinity for C1q among IgG [57], it should be noted that *sIgM*^*-/-*^ mice are known to have steady-state IgG3 level higher than WT mice [46]. In naïve WT mice, however, natural IgM are overwhelmingly the predominant isotype with LPS_*Ft*_-specificity while other isotypes with this specificity, including IgG3, are undetectable. Therefore, it is likely that classical complement activation by LPS_*Ft*_ is predominantly mediated by natural IgM, as previously proposed [30].

The host protection from tularemia is known to depend on both cellular and humoral immune responses and previous studies from our and other groups have shown that IgM_*Ft*_ are protective [38–41]. Our new results indicate that in naïve mice protection from tularemia is mediated primarily by IgM rather than IgG or IgA (Fig 3). However, in immunized mice the opposite is true with IgG3_*Ft*_ playing a more protective role than IgM_*Ft*_. This likely reflects the fact that IgM is the first antibody to be produced during infection while LPS_*Ft*_-immunization protects the host through generation of LPS_*Ft*_-specific B1 memory cells that are class switched. The protective role of IgG3 in bacterial infection is well recognized and it is mediated by complement activation, phagocytosis, and antibody-mediated cytotoxicity [58].

Despite numerous studies demonstrating the crucial role of complement activation in humoral immunity, the extent of its requirement for the response to TI-antigen is less clearly defined. This response has been shown to be affected by several variables, including the antigen’s density, dose, and nature (viral vs. bacterial) [5,52,53,59]. The humoral response against a number of viral TI-2 antigens depends on complement [52]. In contrast, antibody production against NP-ficoll, a classic TI-2 antigen, is complement-independent but does require CR2 [45] (as the results of S2 Fig shows). This suggests that complement activation may not be a universal requirement for TI responses. It is also interesting that in absence of complement certain T-independent antigens become T–dependent [60]. This remains a clearly complex issue.

Anaphylatoxins regulate the immune response directly or indirectly through stimulation of DC, T cells and other cell types [17–19] (reviewed in [20]). However, their role in the response to TI antigens, particularly in B1 B cells, is less well understood. C3a does not trigger DNA synthesis in B cells [61] and it has been shown to suppress human tonsil B cells activation [62]. C3a and C5a have been suggested to indirectly play a positive role in T and B lymphocytes activation by T-dependent antigens through activation of DC [63], a scenario that unlikely can apply to the response to TI antigens. In fact, whether B cells express C3aR1 is controversial [62,64] although both anaphylatoxins receptors are expressed by germinal center B cells and affect the GC response [65]. C5a has been shown to regulate the homeostasis of B1 cells subsets decreasing their number in the peritoneum while increasing them in the spleen [66]. Our results indicate that C3a, but not C5a, plays a critical role for B1 cells’ response to LPS_*Ft*_ by acting on other cell types, likely by stimulating IL-5 synthesis in ILC2 (Fig 7). This result agrees with a report that showed ILC2 activation by C3a [50] and, together with our previous demonstration that B1 cells’ response to LPS_*Ft*_ depended on ILC2-secreted IL-5 [42], it provides a compelling new mechanism for B1 cells activation. It has been shown that *C3ar1*^*-/-*^ mice are more susceptible to *Chlamydia psittaci* infection and had decreased T and B cells activation [67], suggesting an effect on the response against T-dependent antigens. Recently, it has been shown that the response to a synthetic TI antigen depended on alternative complement activation and generation of C3a and C5a [47]. Both anaphylatoxins were then showed to induce BAFF production by neutrophils supporting B cells activation. Our results differ from this report in several aspects: 1) only C3a, but not C5a, was required for B1 cells activation by LPS_*Ft*_ (Fig 4). 2) classical, but not the alternative, complement pathway was involved (Fig 2). 3) neutrophils depletion did not affect B1 cells response to LPS_*Ft*_ immunization (S8 Fig). Therefore, our study shows for the first time that C3a is critically involved in B1 cells activation by a TI-2 bacterial antigen.

It is unclear whether the highly repetitive structure of TI-2 antigens is the sole feature that allows these molecules to activate B1 cells. In the case of LPS_*Ft*_, we showed the necessity of two additional signals generated by complement activation. Future studies will explore the possibility that complement activation is a general requirement that supports B1 cells responses against TI-2 antigens. The relevance of our results for therapeutic interventions should also be examined.

## Materials and methods

### Ethics statement

All the animal experiments were conducted under protocols approved by the Rosalind Franklin University of Medicine and Science Institutional Animal Care and Use Committee (IACUC) (# B23-12, B22-10) as well as by the Italian Ministry of Health (approval no. *543/2025-PR*), in strict accordance with the recommendations in the *Guide for the Care and Use of Laboratory Animals* of the National Institutes of Health. All efforts were made to minimize suffering and ensure the highest ethical and humane standards.

### Mice

C57BL/6J, *Rag1*^*-/-*^*, C3*^*-/-*^*, C5ar1*^*-/-*^*, Aid*^*-/-*^ *(Aicda*^*-/-*^) mice were purchased from Jackson lab. *Cr2*^*-/-*^ mice were obtained from Michael Carrol (Harvard University). *C3ar1*^*-/-*^ from Mark Rothenberg (Cincinnati Children’s Hospital). sIgM^-/-^ (*Ighm*^*tm1Che*^/J) were from Nicole Baumgarth (John Hopkins University). *C1q*^*-/-*^*, C4*^*-/-*^ *mice* were provided by John D. Lambris (University of Pennsylvania). *Mbl1/Mbl2*^*-/-*^, *Cfb*^*-/-*^ and WT mice on a C57BL6/NJ background were purchased from the Jackson Laboratory. All mouse strains were on C57BL/6 genetic background, except *Cfb*^*-/-*^ that were on a C57BL/6NJ genetic background, and were bred under specific pathogen-free conditions in RFU or the Humanitas Research Hospital animal facilities. Experimental groups were composed of 3–13 mice, age-(8–10 weeks old) and sex-matched.

### LPS_*Ft*_ purification, C3d opsonization, and immunoprecipitation

LPS_*Ft*_ was purified from mid logarithmic phase liquid culture of *F. tularensis* LVS using the phenol-based LPS Extraction Kit (iNtRON Biotechnology) as previously described [42]. The LPS preparation was then treated with RNase A, DNase I, and Protienase K, re-extracted, precipitated, and resuspended in Tris NaCl at a concentration of 100 μg/mL. Absence of TLR-agonist contaminants was confirmed by stimulating WT and *Tlr2*^*-/-*^ bone marrow derived macrophages (S9 Fig). For C3d tagging, 300 ng of LPS_*Ft*_ were incubated for 30 minutes at 37°C with 10 μl fresh serum obtained from LPS_*Ft*_-immunized WT mice (this serum provides strong complement activation due to high level of IgM_*Ft*_). An anti-LPS_*Ft*_ monoclonal antibody (FB11, Abcam) was then added. After PAGE and transfer, the PVDF membrane was probed with an anti-C3 antibody (Abcam 200999).

### Mice immunization and treatments

Mice were immunized with 100 ng LPS_*Ft*_ diluted in sterile PBS and administered intraperitoneally (i.p.) (200 μl). For immunization with C3d-opsonized LPS_*Ft*_ 30 μl of fresh serum obtained from LPS_*Ft*_-immunized WT mice were incubated with 100 ng LPS_*Ft*_ for 30 minutes at 37°C. The product was then heat inactivated for 30 minutes at 56°C and used for immunization. For neutrophil depletion, 400 µg of anti-mouse Ly6G 1A8 or isotype control (*InVivo*Mab – BioXcell) were administered i.p. on the day before immunization and then 200 µg on the day of immunization, the following day, and on the third day post immunization).

### Determination of antibody and cytokine levels by ELISA

Blood was collected aseptically from the submandibular vein at the time points indicated in each figure (typically at day 0, 7, 14, 28, 28 + 7, 35, 35 + 7). BALF were collected from euthanized mice by intratracheal injection and aspiration of 1 mL PBS. The peritoneal and thoracic cavities were washed twice with 2 mL PBS/Pen/Strep. LPS_*Ft*_-specific immunoglobulin levels in serum or lavages were measured by ELISA. Serum dilutions were plated in 96 wells plates coated with purified LPS_*Ft*_ (100 ng/mL). HRP-conjugated goat anti-mouse IgM, IgG2b, or IgG_3_ (Southern Biotech Associates, Birmingham, AL) was added followed by TMB substrate and measurement of absorbance at 450 nm. The following dilution were used: IgM_*Ft*_ 1:500 (1:100 *Rag1*^*-/-*^ mice), IgG2b_*Ft*_, IgG3_*Ft*_ 1:50 (1:30 *Rag1*^*-/-*^ mice). Cytokine levels were measured in BALF by ELISA using the following paired antibodies kits: mMCP1, mGM-CSF, mIFN-γ, mIL-1β, mIL-5, mIL-6, mTNFα (Invitrogen). Mouse C5a was measured by ELISA kit (Invitrogen).

### ELISPOT

Multiscreen 96 well Filter plates (Millipore) were coated overnight with LPS_*Ft*_ (50 ng/mL) and blocked in 1% BSA for two hours. Single cell suspensions from spleen, peritoneum, or thoracic cavity were plated (5x10^5^ or 5x10^4^ cells/well) in RPMI1640/10% FCS, Pen/Strep/Amphothericin. Two days later, plates were washed and LPS_*Ft*_-specific spots revealed with HRP-conjugated rat anti-mouse IgM and TMB substrate. Each well was photographed and spots counted.

### Bacteria culture, mice infection, and measurement of bacteria burden in organs

For all experiments the *F. tularensis* LVS strain was used. Bacteria were grown in MH broth (Muller Hinton supplemented with 0.1% glucose, 0.1% cysteine, 0.25% ferric pyrophosphate, and 2.5% calf serum) to mid-logarithmic phase, their titer was determined by plating serial dilutions on complete MH agar, and stocks were maintained frozen at -80°C. No loss in viability was observed over prolonged storage. For infections, frozen stocks were diluted in sterile PBS to the desired titer. Aliquots were plated on complete MH agar to confirm actual cfu. Mice were anesthetized with isoflurane using a Surgivet apparatus and 50 μl of bacteria suspension were applied to the nare. Organs aseptically collected were weighted and homogenized in 1 mL PBS containing 0.5% saponin and 3% BSA. Serial dilutions were plated on complete MH agar plates using the Eddy Jet Spiral Plater (Neutec). Bacterial colonies were counted 48 hours later using the Flash & Grow Automated Bacterial Colony Counter (Neutec).

### Flow cytometry

Single cell suspension obtained from peritoneum cavity lavage or spleen were stained with LIVE/DEAD Yellow fixable viability dye (Invitrogen) and then stained in FACS buffer (1% BSA, 0.05% NaN3 in PBS, 5 µg/mL Fc block CD16/32) with the following anti-mouse antibodies: BV421-conjugated CD19; BV605-conjugated CD23; FITC-conjugated B220; PerCP-Cy5.5-conjugated TCRβ; PE-Dazzle594-conjugated CD43; PE-Cy7-conjugated IgM; APC-conjugated CD21/CD35; Spark NIR 685-conjugated IgD. In the spleen, B1 cells were defined as CD19^Hi^CD43^+^CD23^-^IgM^Hi^IgD^Lo^, FOB cells as CD19^Hi^CD43^-^CD23^+^IgM^Lo^IgD^Hi^ and MZB cells as CD19^Hi^CD43^-^CD23^-^CD21/35^HI^IgM^Hi^IgD^Lo^ [9,68]. In the peritoneal cavity, B1 cells were defined as CD19^Hi^B220^Lo^IgM^Hi^IgD^Lo^ and B2 cells as CD19^Mid^B220^Hi-^IgM^Lo^IgD^Hi^. Data were acquired with a Cytek Aurora (Cytek) and analyzed with FlowJo 10.4 software (Treestar Inc).

### B1 B cells isolation, cell sorting purification, and adoptive transfer

The peritoneal cavity was washed twice with 2 mL PBS/Pen/Strep. B cells were enriched using the Pan B cells kit (StemCells Technologies). B cells (5x10^5^) were adoptively transferred into *Rag1*^*-/-*^ mice by i.p. injection. Four weeks later B1 cells repopulated the peritoneum and mice were used for experiments.

### ILC2 enrichment and *in vitro* culture

Lung was minced and digested with collagenase IV and DNAse I for 45 minutes in RPMI1640/Pen/Strep. Single cell suspension from digested lung was filtered with 70 μm mesh and red blood cells lysed. ILC2 were enriched from cell suspension using EasySep Mouse ILC2 Enrichment Kit (STEMCELL Technologies) and cultured in RPMI1640/10% FCS/Pen/Strep for 18h or 36h, in the presence of 100 ng/mL recombinant murine IL-25 (BioLegend) and/or 2 µg/mL Synthetic C3aR1 receptor agonist (Cayman Chemicals) [50].

### RT-qPCR

Total RNA was isolated from lung homogenate using Trizol. Total RNA (1–2 µg) was use to generate cDNA using random hexamers and SuperScript III First-Strand Synthesis System (Invitrogen). Quantitative PCR was performed using PowerUp SYBR Green (Applied Biosystems) using 1 μl of cDNA template per reaction. The following primers were used: *Il5* (mIL-5) Fwd-TCA GGG GCT AGA CAT ACT GAA G/ Rev-CCA AGG AAC TCT TGC AGG TAA T; *Actb* (β-Actin) Fwd-GGC TGT ATT CCC CTC CAT CG/ Rev-CCA GTT GGT AAC AAT GCC ATG T. Values were calculated through 2-ddCt method for relative fold change in gene expression where ddCt is calculated by subtracting the dCt (gene of interest Ct–bActin Ct) of each experimental sample from the averaged dCt of the calibrator samples (naive mice).

### Statistical analysis

All data were expressed as mean ± SEM. Statistical analysis was performed with GraphPad Prism 7 as specified in figure captions. Mann-Whitney U test was used for all experiments in which we compared, at each time point, two experimental groups. ANOVA or the equivalent non-parametric Kruskal-Wallis test with the appropriate post-hoc test was used for comparison of more than two groups. Gehan-Breslow-Wilcoxon test was used for survival curves. Significance was set at p ≤ 0.05 (*), p ≤ 0.01 (**) and p ≤ 0.001 (***), p ≤ 0.0001 (****).

## Supporting information

S1 Fig Antibody levels of LPS_*Ft*_-specific IgG1, IgE, or IgA were measured in the serum (A), and IgA in the BALF (B), of immunized WT mice at indicated time points. Data are expressed as mean ± SEM. Kruskal-Wallis test with Dunn’s multiple comparison test.
(TIFF)

S2 Fig Antibody levels of NP-ficoll-specific IgM, IgG2b and IgG3 isotypes were measured at the indicated time points in the serum of WT, *C3ar1*^-/-^, *C3*^-/-^ and *Cr2*^-/-^ mice immunized with NP-ficoll (25 μg, i.p.). Data are expressed as mean ± SEM. Kruskal-Wallis test with Dunn’s multiple comparison test. *p* < 0.01**.
(TIFF)

S3 Fig Fresh serum was incubated with Zymosan (0.5 μg), LPS_*Ft*_ (33 ng), NP-ficoll (1 μg) or LPS_*Fn*_ (33 ng) and generation of C5a was measured by ELISA. One-way ANOVA with Tukey’s multiple comparison test. *p* < 0.0001****.
(TIFF)

S4 Fig Total B1 cells numbers in peritoneal cavity or spleen of the indicated mouse strains at shown time points. Data are expressed as mean ± SEM. Two-way ANOVA with Tukey’s multiple comparison test. *p* < 0.05*, *p* < 0.0001****.
(TIFF)

S5 Fig Lung inflammation in infected WT, *C3ar1*^*-/-*^, or *C5ar1*^*-/-*^ mice. Wild type or *C3*^*-/-*^*, C5ar1*^*-/-*^, or *C3ar1*^*-/-*^ mice were infected with *Ft* LVS (5.5x10^3^ cfu) and cytokine levels in BALF (A) and lung myeloid cells infiltration (B) were measured 7 days after infection. One representative experiment of 2. Data are expressed as mean ± SEM. One-way ANOVA with Dunnett’s multiple comparison test. *p* < 0.05*, *p* < 0.01**, *p* < 0.001***.
(TIFF)

S6 Fig Antibody levels were measured in serum of immunized WT, *C3ar1*^*-/-*^ or *Cr2*^*-/-*^ mice (of Fig 5D) at indicated time points before infection with *Ft* LVS. Data are expressed as mean ± SEM. Kruskal-Wallis test with Dunn’s multiple comparison test. *p* < 0.05*, *p* < 0.01**.
(TIFF)

S7 Fig Total B1 cells numbers in peritoneal cavity or spleen of the indicated mouse strains 28 days after reconstitution. Data are expressed as mean ± SEM. Two-way ANOVA with Tukey’s multiple comparison test. *p* < 0.05*, *p* < 0.01**.
(TIFF)

S8 Fig Neutrophil depletion does not impair B1 cells’ response to immunization with LPS_*Ft*_. WT mice were treated with anti-mouse Ly6G 1A8 or isotype control and immunized with LPS_*Ft*_. Antibody levels were measured in serum at indicated time points. Data are expressed as mean ± SEM. Kruskal-Wallis test with Dunn’s multiple comparison test. *p* < 0.05*.
(TIFF)

S9 Fig WT and *Tlr2*^*-/-*^ bone marrow derived macrophages were stimulated for 18h with 100 ng/mL LPS from E. coli 0111:B4, 100 ng/mL LPS_*Ft*_, or 2 μg/mL PAM3Cys. Secreted TNFα and IL-6 were measured by ELISA in the culture supernatant. Data are expressed as mean ± SEM. Two-way ANOVA with Dunnett’s multiple comparison test. *p* < 0.0001****.
(TIFF)

S1 Data Raw data for Fig 1 in pzfx format. (PZFX)

S2 Data Raw data for Fig 2 in pzfx format. (PZFX)

S3 Data Raw data for Fig 3 in pzfx format. (PZFX)

S4 Data Raw data for Fig 4 in pzfx format. (PZFX)

S5 Data Raw data for Fig 5 in pzfx format. (PZFX)

S6 Data Raw data for Fig 6 in pzfx format. (PZFX)

S7 Data Raw data for Fig 7 in pzfx format. (PZFX)

S8 Data Raw data for S1–S9 Figs in pzfx format. (PZFX)
