# Supplementary figures and images for: Activation of B1 B cells by F. tularensis atypical LPS depends on classical complement and C3a
Source: PLoS Pathog. 2025 Dec 17;21(12):e1013799. doi: 10.1371/journal.ppat.1013799 (PMC12725618; doi:10.1371/journal.ppat.1013799)

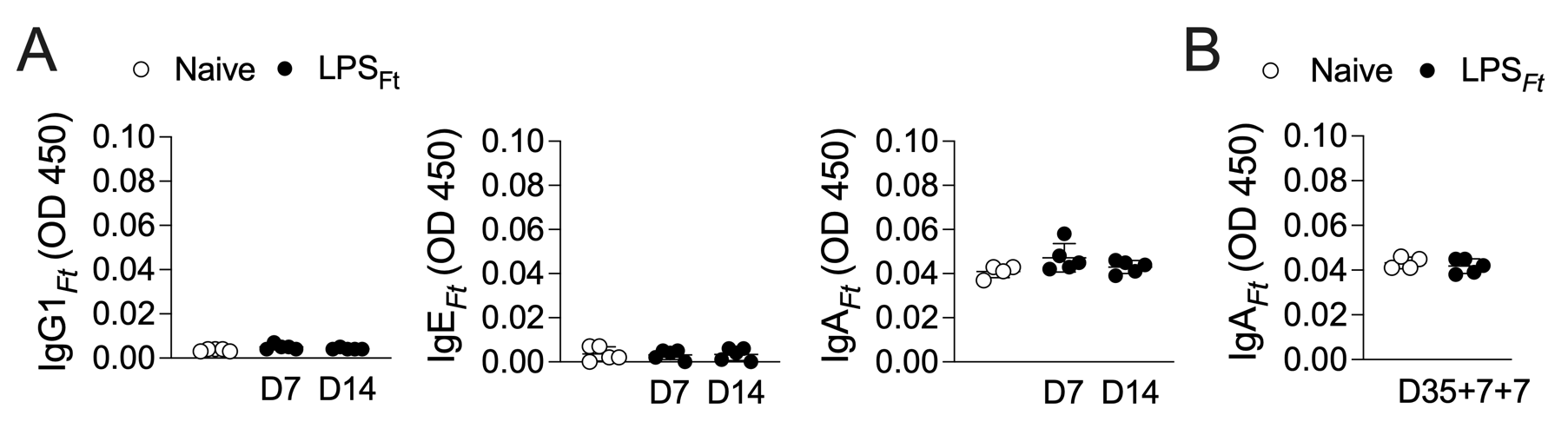

Supplement: S1 Fig — Data are expressed as mean ± SEM. Kruskal-Wallis test with Dunn’s multiple comparison test. (TIFF) [file ppat.1013799.s001.tiff]

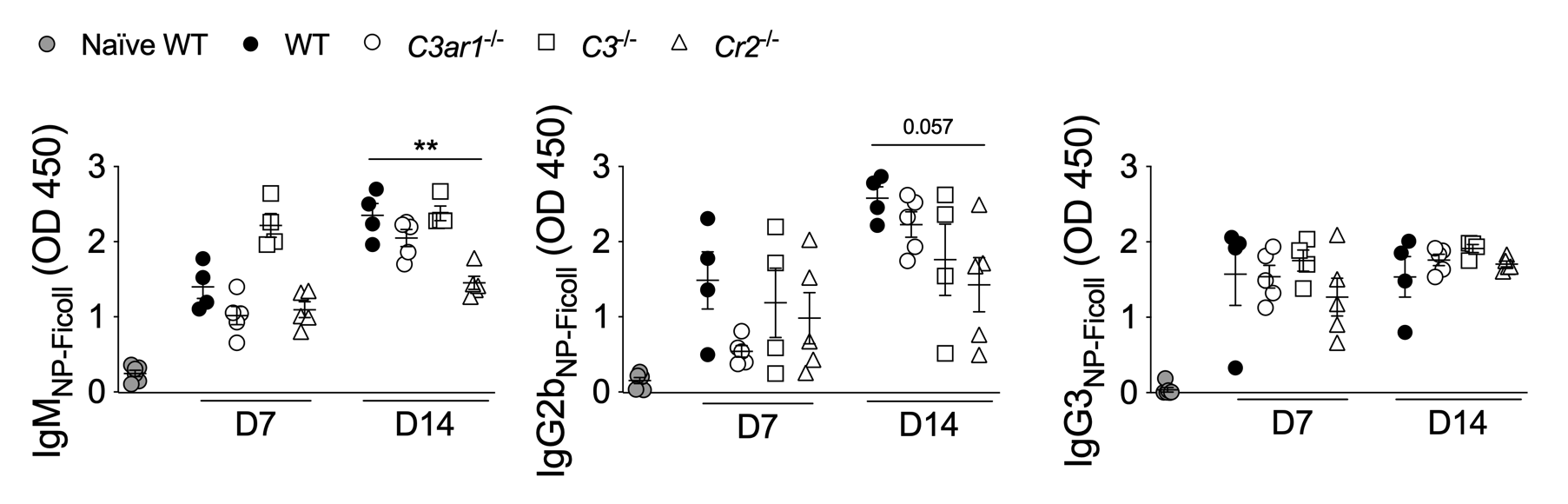

Supplement: S2 Fig — Data are expressed as mean ± SEM. Kruskal-Wallis test with Dunn’s multiple comparison test. p < 0.01**. (TIFF) [file ppat.1013799.s002.tiff]

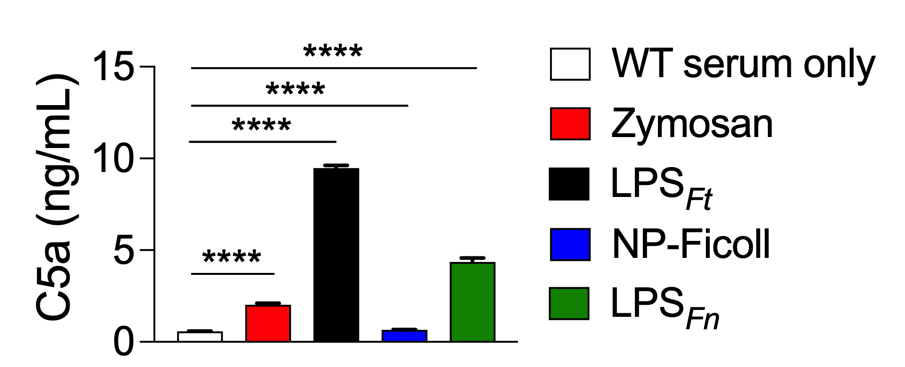

Supplement: S3 Fig — One-way ANOVA with Tukey’s multiple comparison test. p < 0.0001****. (TIFF) [file ppat.1013799.s003.tiff]

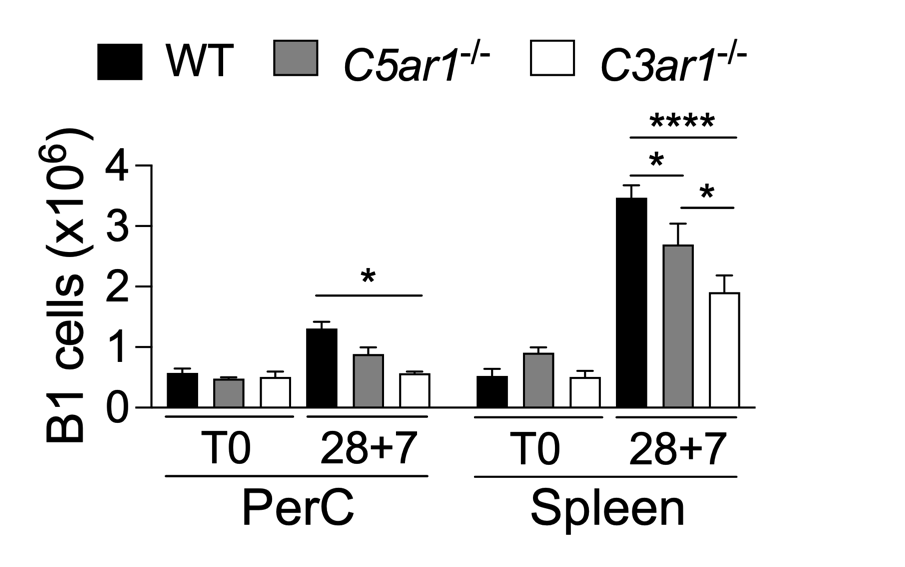

Supplement: S4 Fig — Data are expressed as mean ± SEM. Two-way ANOVA with Tukey’s multiple comparison test. p < 0.05*, p < 0.0001****. (TIFF) [file ppat.1013799.s004.tiff]

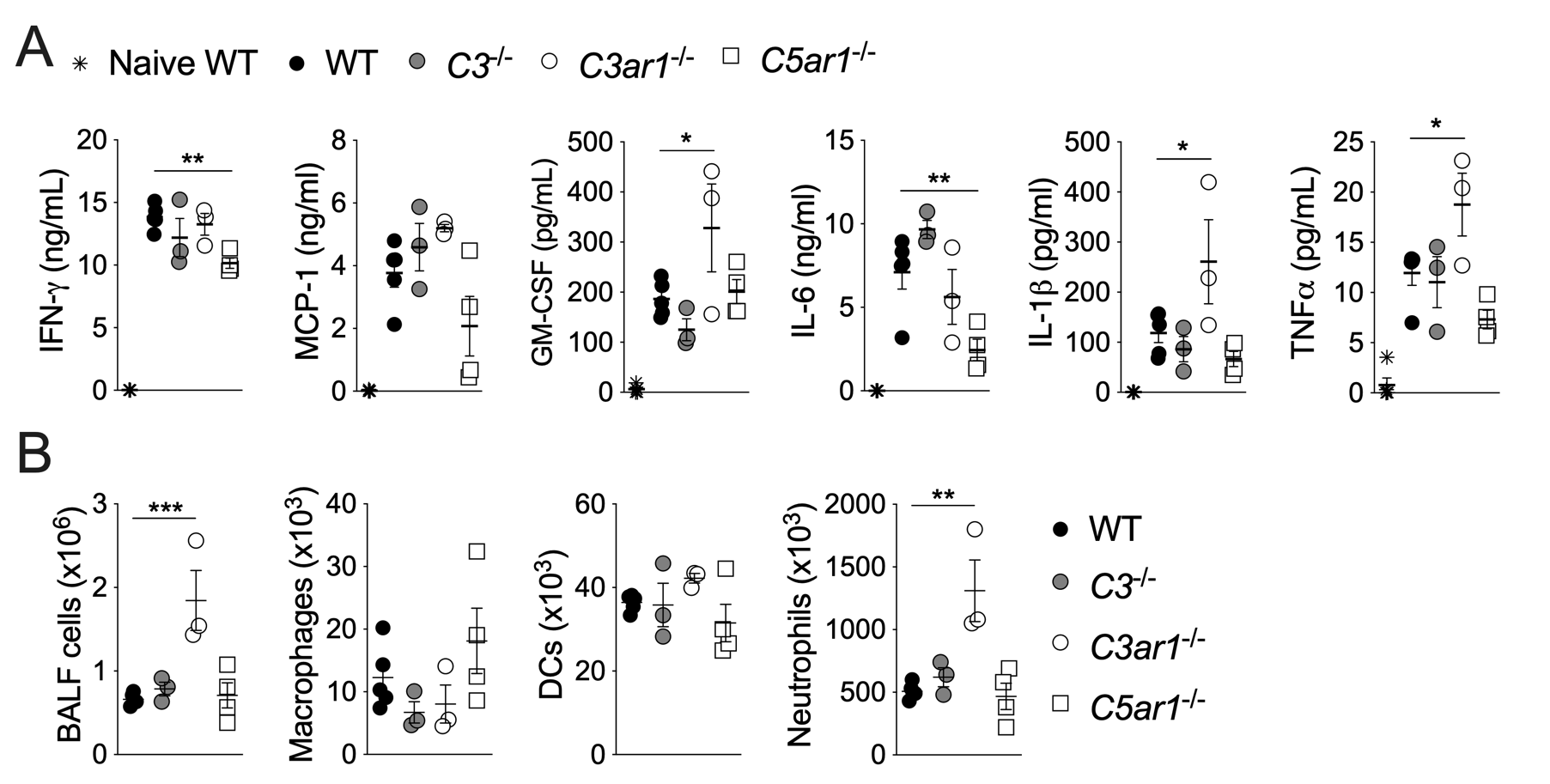

Supplement: S5 Fig — Wild type or C3-/-, C5ar1-/-, or C3ar1-/- mice were infected with Ft LVS (5.5x10^3^ cfu) and cytokine levels in BALF (A) and lung myeloid cells infiltration (B) were measured 7 days after infection. One representative experiment of 2. Data are expressed as mean ± SEM. One-way ANOVA with Dunnett’s multiple comparison test. p < 0.05*, p < 0.01**, p < 0.001***. (TIFF) [file ppat.1013799.s005.tiff]

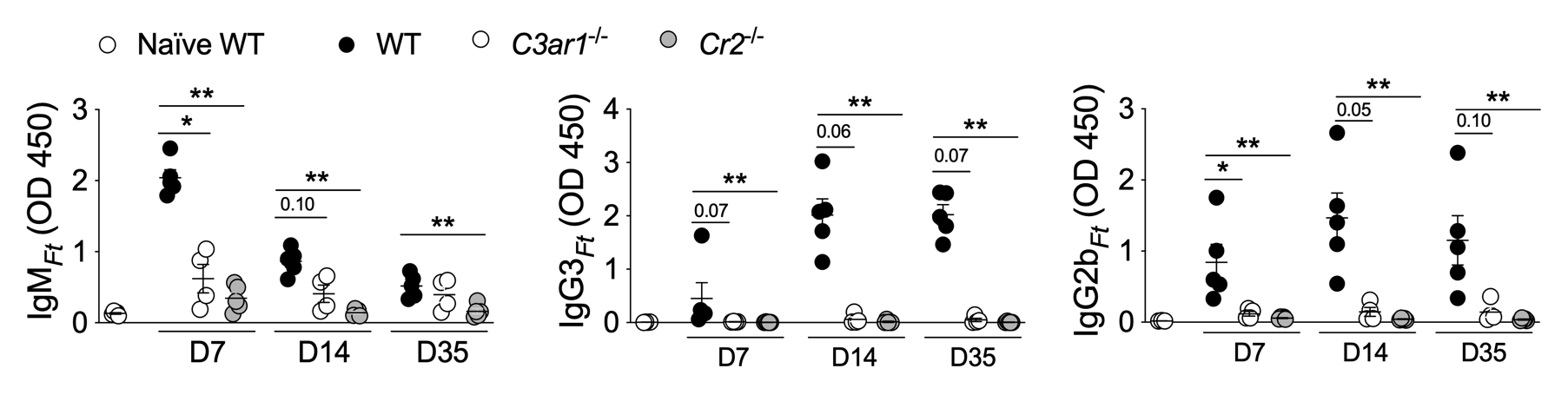

Supplement: S6 Fig — Data are expressed as mean ± SEM. Kruskal-Wallis test with Dunn’s multiple comparison test. p < 0.05*, p < 0.01**. (TIFF) [file ppat.1013799.s006.tiff]

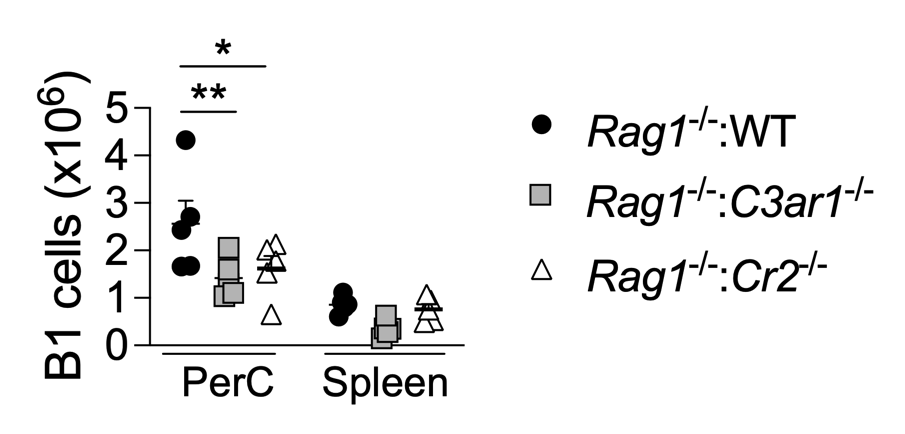

Supplement: S7 Fig — Data are expressed as mean ± SEM. Two-way ANOVA with Tukey’s multiple comparison test. p < 0.05*, p < 0.01**. (TIFF) [file ppat.1013799.s007.tiff]

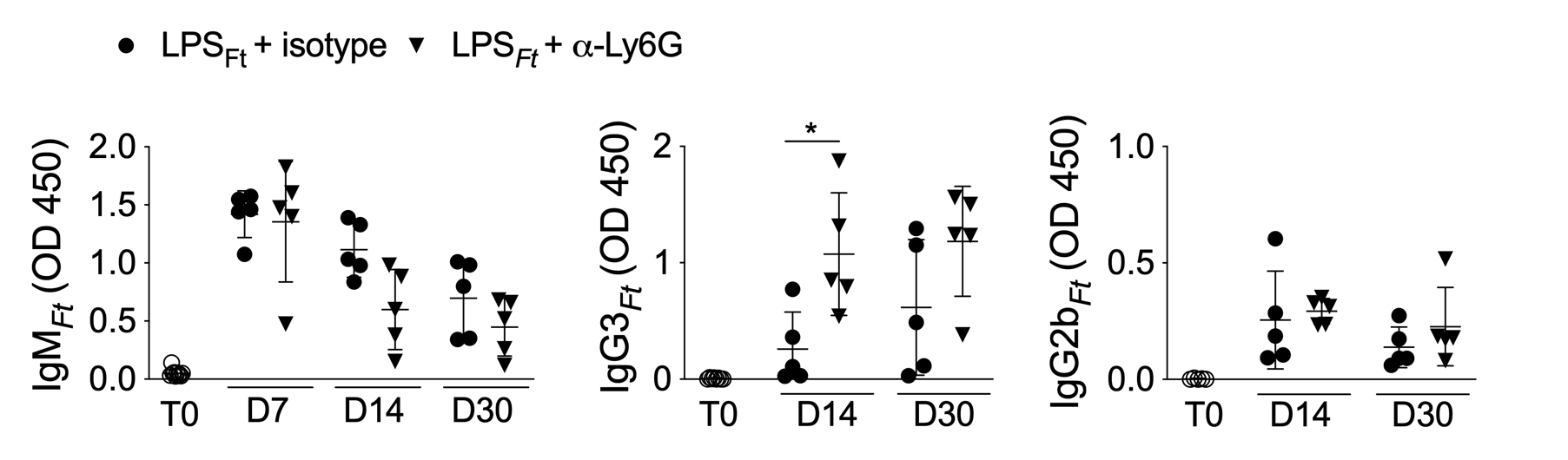

Supplement: S8 Fig — Data are expressed as mean ± SEM. Kruskal-Wallis test with Dunn’s multiple comparison test. p < 0.05*. (TIFF) [file ppat.1013799.s008.tiff]

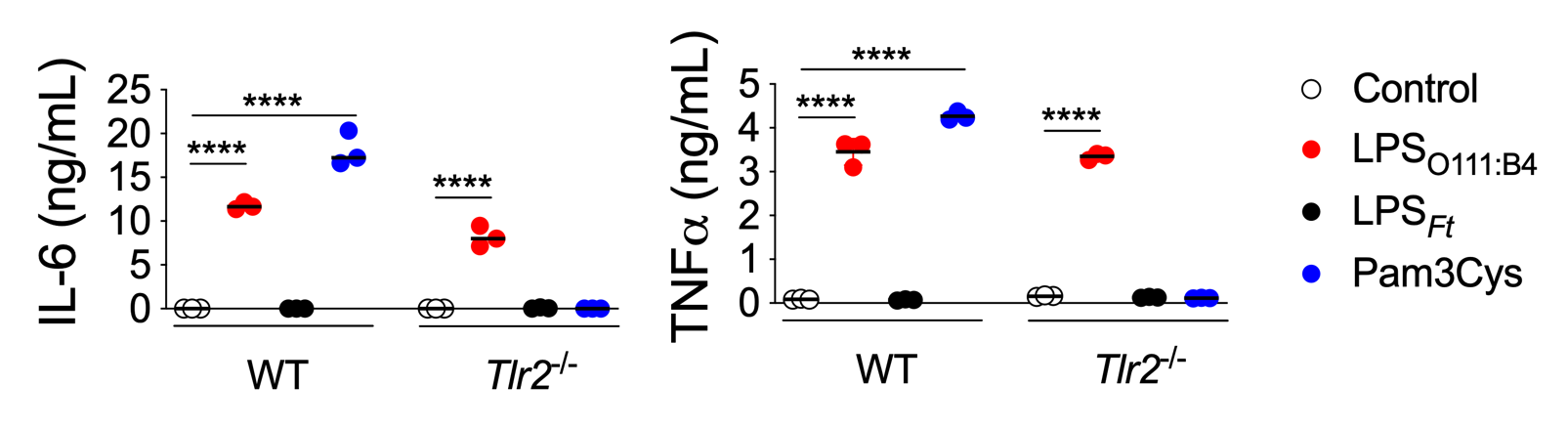

Supplement: S9 Fig — Secreted TNFα and IL-6 were measured by ELISA in the culture supernatant. Data are expressed as mean ± SEM. Two-way ANOVA with Dunnett’s multiple comparison test. p < 0.0001****. (TIFF) [file ppat.1013799.s009.tiff]
